# Sleeping <6.55 h per day was associated with a higher risk of low back pain in adults aged over 50 years: a Korean nationwide cross-sectional study

**DOI:** 10.3389/fpubh.2024.1429495

**Published:** 2024-09-20

**Authors:** Dexin Hu, Yihui Zhang, Xingkai Liu, Xin Yang, Xichao Liang, Xu Hu, Hua Yuan, Chenguang Zhao

**Affiliations:** ^1^Department of Rehabilitation Medicine, Xijing Hospital, Fourth Military Medical University, Xi'an, Shaanxi, China; ^2^School of Sports Medicine and Rehabilitation, Beijing Sports University, Beijing, China

**Keywords:** low back pain, sleep duration, cross-sectional analysis, KNHANES, Korean older adult

## Abstract

**Background:**

Patients with low back pain (LBP) often suffer from sleep disorder, and insufficient sleep duration was recognized as a potential risk factor for LBP. Our aim was to explore the exact effect of sleep duration on LBP and the optimal sleep duration to reduce the risk of LBP.

**Methods:**

Analyzing data from the Korean National Health and Nutrition Examination Survey (KNHANES), we investigated the association between sleep duration and LBP in individuals aged 50 years and older. We used logistic regression models, interaction stratification analysis, and threshold effect assessment to analyze the relationship between sleep duration and LBP.

**Results:**

A total of 6,285 participants, comprising 3,056 males and 3,229 females with a median age of 63.1 years, were enrolled in the study. The association between sleep duration and LBP risk exhibited an L-shaped curve (*p* < 0.015) in RCS analysis. In the threshold analysis, the OR of developing risk of LBP was 0.864 (95% CI:0.78–0.957, *p* = 0.005) in participants with sleep duration <6.55 h. Each additional hour of sleep was associated with a 13.6% decrease in the risk of LBP. No significant association was observed between sleep duration ≥6.55 h and the risk of LBP. The risk of LBP did not decrease further with increasing sleep duration. Results remain robust across subgroups.

**Conclusion:**

Our findings indicate that shorter sleep duration is a risk factor for LBP in adults aged over 50 years. We revealed an L-shaped association between sleep duration and LBP, with an inflection point at approximately 6.55 h per day. These results underscore the significance of sleep duration as a factor in the risk assessment for LBP.

## 1 Introduction

Low back pain (LBP) is a significant contributor to the global burden of disability (1). Based on data from the most recent Global Burden of Diseases, Injuries, and Risk Factors Study (GBD), the estimated global prevalence of individuals with LBP in 2020 was 619 million people (2). It is projected that by 2050, the number of affected individuals will increase to 843 million. Meanwhile the GBD data has revealed a marked rise in the occurrence of LBP within the Asian population. It is to be expected that both the burden of disability and the costs associated with LBP will increase further (3).

Previous research on LBP has largely focused on exploring the pathophysiological causes at the biomedical level, such as herniated discs, degenerative spinal deformity, sprain, and spinal stenosis (4, 5). However, the etiology of LBP involves multidimensional factors including biological, sociological, and psychological aspects (6, 7). Evidence suggests that gender, type of strength training, body mass index (BMI), affective states of depression, and the presence of social support are hidden risk factors for LBP (8, 9). To optimize the management and prevention of LBP, a detailed comprehension of the relationship between key factors and LBP is necessary (10).

Sleep is essential for survival (11). While asleep, the body orchestrates a multitude of vital functions, including the regulation of blood pressure and heart rate, hormonal secretion, immune system efficacy, cellular repair, thermoregulatory balance, the recovery of memory capabilities, and the enhancement of cognitive processes (12). However, sleep can also be marred by a variety of sleep disorders or abnormal behaviors, including insomnia, obstructive sleep apnea, circadian rhythm sleep-wake disorders, and sleep bruxism, which can significantly impact overall health and wellbeing (13, 14). Previous studies revealed a significant correlation between sleep and pain (15). Sleep disorders can lead to increased perception of pain severity and may negatively influence the patient's return to functional capabilities (16). Epidemiological studies have revealed a high prevalence of sleep disorders among LBP patients, with more than 50% affected, and have demonstrated a substantial negative correlation between sleep disorders and LBP (17, 18). The incidence of LBP is significantly associated with decreased sleep duration, deterioration in sleep quality, intensified difficulty initiating sleep, decline in daytime functionality, and an increase in sleep dissatisfaction and distress (19). The predominant focus in LBP management is on pain intensity and disability (20). However, the latest review indicates that the evaluation of sleep conditions has been neglected in many clinical research studies of LBP, an oversight that limits our understanding of the interplay between sleep-related factors and the occurrence of LBP (21).

Considering the relevant background, our study utilizes a nationally representative sample of Korean adults to achieve the following aims: (1) determine whether sleep duration is an independently associated risk factor for LBP; (2) investigate the dose-response relationship between sleep duration and LBP. The findings may help to clarify the role of sleep duration as a risk factor for LBP and provide information for future LBP management strategies in the general population.

## 2 Materials and methods

### 2.1 Study design and participants

This is a population-based cross-sectional study conducted using nationally representative survey data, all of which were publicly obtained from The Korea National Health and Nutrition Examination Survey (KNHANES) website (https://knhanes.kdca.go.kr/knhanes/eng/index.do). The KNHANES is an annual nationwide survey organized and executed by the Korea Centers for Disease Control and Prevention (KCDC) since 1998. With the assistance of experienced interviewers, healthcare personnel, and laboratory professionals, the KNHANES systematically collects a comprehensive array of health-related data through the administration of health interviews, health examinations, and nutritional assessments on participants. This approach allows for the evaluation of health and nutritional conditions and trends among South Korean residents. In the survey, participants were selected utilizing probability-sampling techniques, employing a multistage clustering approach that considered age group, region, and gender extracted from household registries (22).

**Figure 1:**
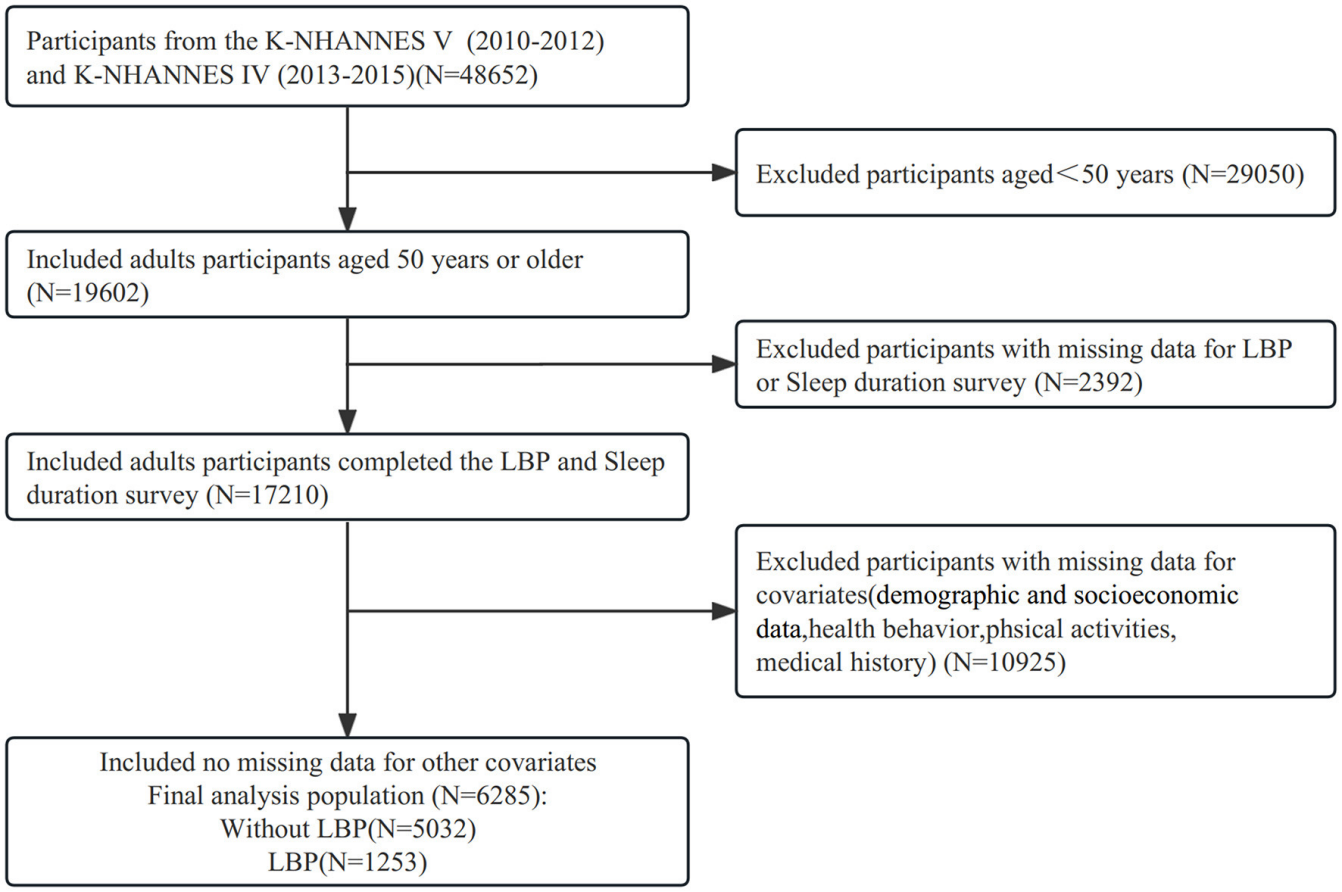
Flow diagram of study participants. The figure represents the information of excluded participants based on 4,8652 participants. A total of 6,285 participants with complete information at baseline were eligible for the cross-sectional analysis. K-NHANES, Korea National Health and Nutrition Examination Survey; LBP, Low Back Pain.

The study sample consisted of 48,652 participants from the KNHANES-V and VI surveys conducted from 2010 to 2015. The study focused on individuals aged 50 years and older, as participants below the age of 50 were not examined for LBP in the survey. Furthermore, individuals with missing data on LBP, sleep duration, and relevant covariates were excluded from this study. In total, 6,285 participants with complete data were enrolled in this study. The participant inclusion and exclusion process was illustrated in Figure 1.

### 2.2 Exposed variables and outcome variables

Participants were considered to experience LBP if they responded affirmatively to the following query during survey: “Have you reported experiencing LBP persisting for a duration of 30 days or longer within the most recent 3-month period (22).”

In the KNHANES, the evaluation of habitual total sleep duration, which includes daytime napping, was obtained through the following inquiry: “How many hours do you typically sleep per day?” Participants' responses were recorded using an integer scale. The International Classification of Sleep Disorders classifies a “short sleeper” as an individual who experiences a sleep duration of under 5 h, and a “long sleeper” as an individual who experiences a sleep duration exceeding 9 h (13). Consequently, study participants were divided into five categories: ≤ 5, 6, 7, 8, and ≥9 h.

### 2.3 Description of related variables

The covariates utilized in this study for analysis were gathered through health interview and examination. We focused on variables that had been previously correlated with LBP and sleep duration in the existing literature (2, 23–28). These variables included demographic characteristics (age, sex, height, weight, and BMI), socioeconomic status (household income, educational level, and occupation), health-related behaviors (smoking and alcohol consumption, time spent on walking, resistance training, and flexibility exercises), psychological aspects (psychological stress and depression), and comorbidities (seven other diseases, such as hypertension).

Detailed descriptions of these specific variables are provided below. The household income level was quartile-categorized as: low, low-moderate, moderate-high, and high. Educational attainment was stratified into four categories based on the highest degree achieved: elementary school, middle school, high school, and university or college. Participants' current occupational status was classified into five distinct groups: office workers (e.g., professionals, office workers, and managers), sales and services, agriculture, forestry and fishery, machine fitting and simple labor (e.g., technicians, low-level laborers, and device and machine operators), unemployed (e.g., housewives and students) (29, 30). Within the domain of health behaviors, three variables associated with physical activity, including weekly time allocation for walking, resistance training, and flexibility exercises, were utilized. These variables were uniformly categorized into four groups: none, 1–2 days per week, 3–4 days per week, and ≥5 days per week (26, 28). The classification of smoking habits among participants was simplified into two categories: current and non-/ex-smoker. Additionally, drinking habits were classified based on the frequency of alcohol intake over the past year: none, ≤ 1 drink/month, 2 drinks/month to 3 drinks/week, and ≥4 drinks/week. Psychological health status primarily encompasses subjective stress level and depressive conditions. The former evaluates participants' self-reported psychological stress intensity in their daily lives, categorized as severe, moderate, mild, or none (27). The latter assesses whether participants have been diagnosed with depression by a medical professional (24). Comorbid conditions, including hypertension, diabetes, dyslipidemia, stroke, myocardial infarction, angina, and arthritis, were also determined through medical diagnosis.

### 2.4 Statistical analysis

A logistic regression of a binary response variable (LBP) on a continuous, normally distributed variable (sleep duration) with a sample size of 6,285 observations achieves 85% power at a 0.05000 significance level to detect a change in the Probability of LBP being positive from a value of 0.199 at the mean of sleep duration to 0.183 when sleep duration is increased to one standard deviation above the mean. This change corresponds to an odds ratio of 0.900. An adjustment was made since a multiple regression of the independent variable of interest on the other independent variables in the logistic regression obtained an R-Squared of 0.200. The statistical analyses in this study utilized the R statistical software package (http://www.R-project.org, R Foundation) along with Free Statistics Software versions 1.8. Categorical variables were represented as numbers (percentages), whereas continuous variables were expressed as mean ± standard deviation. Statistically significant results were defined as two-tailed *p*-values <0.05.

Undertaking a comprehensive descriptive analysis to meticulously assess the characteristics of the included participants. This multifaceted analysis compared the basic characteristics of subjects grouped by different sleep durations, as well as those of participants with and without LBP. The specific analytical techniques employed encompass the utilization of chi-square tests for assessing categorical variables and the application of one-way analysis of variance or Student's *t-*test for the evaluation of continuous variables.

Utilizing logistic regression analysis, we assessed the relationship between sleep duration and LBP, subsequently calculating the corresponding odds ratios (ORs) and their associated 95% confidence intervals (CIs). In order to eliminate the possibility of differences attributed to sleep duration from being confounded by potential variables, we systematically adjusted for covariates through the utilization of three distinct logistic regression models. Model 1 was adjusted to account for essential demographic variables, including age and gender. This adjustment was justified by the findings of studies conducted by Jennifer and colleagues, which indicated a correlation between age, sex, and an elevated risk of LBP (31). In Model 2, we performed adjustments for covariates whose effect estimates were altered by more than 10% upon their inclusion in the model. These covariates included age, sex, household income level, educational level, height, weight, self-perceived stress level, and a history of osteoarthritis diagnosis. Beyond the variables adjusted in Model 2, Model 3 executed a comprehensive adjustment, encompassing supplementary variables like occupation, smoking status, alcohol consumption, depressive symptoms, different categories of physical exercise (walking, resistance training, and flexibility exercises) duration, and additional comorbidities. In the process of stepwise adjustments, sleep duration was categorized and sensitivity analyses were performed to evaluate the stability of the findings.

A trend test was subsequently applied to Model 3 to evaluate the presence of a linear trend in the relationship between sleep duration, considered as a continuous variable, and LBP. Dose-response curve between sleep duration and LBP were analyzed using restricted cubic spline (RCS) regression on the 5^th^, 35^th^, 65^th^, and 95^th^ percentiles of sleep duration. Upon detection of a non-linear relationship, a two-piecewise linear logistic regression model was applied to investigate the association threshold between sleep duration and LBP after adjusting the potential confounders in Model 3. Additionally, a recurrence method was utilized to define the threshold value for sleep duration, including the selection of turning points along a predefined interval and the choice of the turning point resulting in the maximum likelihood model.

Lastly, within discrete strata defined by age, gender, household income, educational attainment, and occupation, stratified logistic regression models were employed for the purpose of conducting subgroup analyses. To test interactions across these subgroups, the likelihood ratio test was applied.

## 3 Results

### 3.1 Characteristics of the study population according to low back pain

In this study, 48,652 potential participants were selected from KNHANES (2010–2015), of which 19,602 adults (≥50 years) completed health interviews for inclusion in our study. Participants with missing LBP and sleep duration (*n* = 2,392) were excluded. After further excluding those with missing covariate information (*n* = 10,925), the final analytical sample comprised 6,285 participants (48.6% male and 51.8% female). The average age and sleep duration of the participants are 63.1 ± 8.7 years and 6.6 ± 1.5 h. The baseline characters of the population included and excluded are presented in Supplementary Table S1.

Table 1 presents a comparative analysis between 1,253 LBP patients and 5,032 subjects without LBP. Compared to the group without LBP, LBP patients are obviously older (65.9 ± 8.9 years old vs. 62.5 ± 8.5 years old; *p* < 0.001) and have shorter sleep duration (6.4 ± 1.7 h vs. 6.7 ± 1.4 h; *p* < 0.001). Besides, females, a lower household income, and a lower education level were combined with an increased risk of LBP. The Supplementary Table S2 describes in detail the baseline characteristics of the subjects stratified by distinct sleep duration categories.

**Table 1:** KNHANES 2010–2015 participant characteristics stratified by LBP status.

**Variables**	**Total (*n =* 6,285)**	**Without LBP (*n =* 5,032)**	**With LBP (*n =* 1,253)**	***p***
Sleep duration (hour), Mean ± SD	6.6 ± 1.5	6.7 ± 1.4	6.4 ± 1.7	<0.001
Age (year), Mean ± SD	63.1 ± 8.7	62.5 ± 8.5	65.9 ± 8.9	<0.001
Height (cm), Mean ± SD	160.4 ± 8.7	161.2 ± 8.5	156.9 ± 8.5	<0.001
Weight (kg), Mean ± SD	61.9 ± 10.2	62.6 ± 10.2	59.5 ± 9.6	<0.001
Body mass index (kg/m^2^), Mean ± SD	24.0 ± 3.1	24.0 ± 3.1	24.1 ± 3.1	0.288
Sex, *n* (%)				<0.001
Male	3,056 (48.6)	2,659 (52.8)	397 (31.7)	
Female	3,229 (51.4)	2,373 (47.2)	856 (68.3)	
**Household income**, ***n*** **(%)**				<0.001
Low	1,693 (26.9)	1,141 (22.7)	552 (44.1)	
Low-mid	1,704 (27.1)	1,392 (27.7)	312 (24.9)	
Mid-high	1,423 (22.6)	1,231 (24.5)	192 (15.3)	
High	1,465 (23.3)	1,268 (25.2)	197 (15.7)	
**Education level**, ***n*** **(%)**				<0.001
Elementary school	2,504 (39.8)	1,780 (35.4)	724 (57.8)	
Middle school	1,151 (18.3)	936 (18.6)	215 (17.2)	
High school	1,677 (26.7)	1,459 (29)	218 (17.4)	
College or university	953 (15.2)	857 (17)	96 (7.7)	
**Occupation**, ***n*** **(%)**				<0.001
Office work	638 (10.2)	579 (11.5)	59 (4.7)	
Sales and services	655 (10.4)	557 (11.1)	98 (7.8)	
Agriculture, forestry and fishery	495 (7.9)	405 (8)	90 (7.2)	
Machine fitting and simple labor/manual labor	1,460 (23.2)	1,259 (25)	201 (16)	
Unemployed (student, housewife, etc.)	3,037 (48.3)	2,232 (44.4)	805 (64.2)	
**Walking**, ***n*** **(%)**				<0.001
None	1,293 (20.6)	978 (19.4)	315 (25.1)	
1–2 day/week	939 (14.9)	754 (15)	185 (14.8)	
3–4 day/week	1,257 (20.0)	1,010 (20.1)	247 (19.7)	
≥5 day/week	2,796 (44.5)	2,290 (45.5)	506 (40.4)	
**Resistance training**, ***n*** **(%)**				<0.001
None	4,749 (75.6)	3,705 (73.6)	1,044 (83.3)	
1–2 day/week	497 (7.9)	418 (8.3)	79 (6.3)	
3–4 day/week	444 (7.1)	372 (7.4)	72 (5.7)	
≥5 day/week	595 (9.5)	537 (10.7)	58 (4.6)	
**Flexibility exercises**, ***n*** **(%)**				<0.001
None	2,849 (45.3)	2,213 (44)	636 (50.8)	
1–2 day/week	907 (14.4)	743 (14.8)	164 (13.1)	
3–4 day/week	986 (15.7)	780 (15.5)	206 (16.4)	
≥5 day/week	1,543 (24.6)	1,296 (25.8)	247 (19.7)	
**Smoking status**, ***n*** **(%)**				0.095
Non/ex-smoker	5,181 (82.4)	4,128 (82)	1,053 (84)	
Current smoker	1,104 (17.6)	904 (18)	200 (16)	
**Alcohol consumption**, ***n*** **(%)**				<0.001
None	1,611 (25.6)	1,221 (24.3)	390 (31.1)	
≤ 1 drink/month	1,982 (31.5)	1,545 (30.7)	437 (34.9)	
2 drinks/month to 3 drinks/week	2,056 (32.7)	1,741 (34.6)	315 (25.1)	
≥4 drinks/week	636 (10.1)	525 (10.4)	111 (8.9)	
**Degree of Stress**, ***n*** **(%)**				<0.001
None	1,539 (24.5)	1,303 (25.9)	236 (18.8)	
Mild	3,590 (57.1)	2,930 (58.2)	660 (52.7)	
Moderate	927 (14.7)	643 (12.8)	284 (22.7)	
Severe	229 (3.6)	156 (3.1)	73 (5.8)	
**Depression**, ***n*** **(%)**				<0.001
No	5,897 (93.8)	4,799 (95.4)	1,098 (87.6)	
Yes	388 (6.2)	233 (4.6)	155 (12.4)	
**Hypertension**, ***n*** **(%)**				<0.001
No	3,983 (63.4)	3,267 (64.9)	716 (57.1)	
Yes	2,302 (36.6)	1,765 (35.1)	537 (42.9)	
**Diabetes**, ***n*** **(%)**				<0.001
No	5,349 (85.1)	4,340 (86.2)	1,009 (80.5)	
Yes	936 (14.9)	692 (13.8)	244 (19.5)	
**Dyslipidemia**, ***n*** **(%)**				<0.001
No	4,874 (77.5)	3,978 (79.1)	896 (71.5)	
Yes	1,411 (22.5)	1,054 (20.9)	357 (28.5)	
**Stroke**, ***n*** **(%)**				<0.001
No	6,005 (95.5)	4,849 (96.4)	1,156 (92.3)	
Yes	280 (4.5)	183 (3.6)	97 (7.7)	
**Myocardial infarction**, ***n*** **(%)**				0.316
No	6,180 (98.3)	4,952 (98.4)	1,228 (98)	
Yes	105 (1.7)	80 (1.6)	25 (2)	
**Angina**, ***n*** **(%)**				<0.001
No	6,100 (97.1)	4,905 (97.5)	1,195 (95.4)	
Yes	185 (2.9)	127 (2.5)	58 (4.6)	
**Arthritis**, ***n*** **(%)**				<0.001
No	5,104 (81.2)	4,293 (85.3)	811 (64.7)	
Yes	1,181 (18.8)	739 (14.7)	442 (35.3)	

SD, standard deviation.

### 3.2 Association between sleep duration and low back pain

Table 2 summarizes the results of the multivariate logistical regression analysis for the relationship between sleep duration and LBP. The crude model suggested that sleep duration negatively correlated to the occurrence of LBP (OR = 0.87, 95% CI: 0.84–0.91). This means that a 1-h sleep duration increase was linked to a 13% lower risk of LBP. Moreover, we considered sleep duration as a categorical variable (≤ 5,6,7,8, ≥9 h) and found that participants who had 6, 7, 8, ≥9 h of sleep duration had a reduced risk of LBP compared with “short sleepers” who had sleep duration ≤ 5 h. Comparable results were noted upon adjustment for age and sex within model 1 (OR = 0.92; 95% CI: 0.88–0.95). After progressive adjustment for potential confounding factors, the odds ratios from model 3 still indicated a significant inverse relationship between sleep duration and the incidence of LBP. In the fully adjusted Model 3, subjects who slept 7 to 8 h per night had a reduced risk of LBP compared to those who slept 5 h or less, referred to as “short sleepers.”

**Table 2:** Logistic regression to assess the relationship between sleep duration and LBP.

**Variable**	**n. total**	**n. LBP_%**	**OR (95% CI)**							
			**Non-adjusted**	***p*** **-value**	**Model 1**	***p*** **-value**	**Model 2**.	***p*** **-value**	**Model 3**.	***p*** **-value**
Sleep duration (hour)	6,285	1,253 (19.9)	0.87 (0.84~0.91)	<0.001	0.92 (0.88~0.95)	<0.001	0.95 (0.91~0.99)	0.011	0.94 (0.9~0.98)	0.004
Q1 (≤ 5h)	1,336	374 (28)	1(Ref)		1(Ref)		1(Ref)		1(Ref)	
Q2 (6h)	1,635	304 (18.6)	0.59 (0.49~0.7)	<0.001	0.73 (0.61~0.88)	0.001	0.83 (0.69~1)	0.045	0.84 (0.69~1.01)	0.069
Q3 (7h)	1,592	257 (16.1)	0.5 (0.41~0.59)	<0.001	0.62 (0.51~0.75)	<0.001	0.76 (0.62~0.92)	0.005	0.77 (0.63~0.94)	0.01
Q4 (8h)	1,286	217 (16.9)	0.52 (0.43~0.63)	<0.001	0.66 (0.55~0.81)	<0.001	0.78 (0.64~0.96)	0.019	0.8 (0.65~0.99)	0.036
Q5 (≥9h)	436	101 (23.2)	0.78 (0.6~1)	0.048	0.84 (0.65~1.09)	0.191	0.88 (0.67~1.15)	0.349	0.8 (0.6~1.05)	0.109
Trend. test	6,285	1,253 (19.9)		<0.001		0.001		0.043		0.023

Model 1: Adjusted for age, sex. Model 2: Adjusted for the variables in Model 1 plus household income, education level, height, weight, self-reported stress level, Arthritis. Model 3: Adjusted for the variables in Model 2 plus occupation, body mass index, smoking, alcohol consumption, time spent on walking, resistance training, and flexibility exercises, depression, comorbidities (such as hypertension, diabetes, dyslipidemia, stroke, myocardial infarction, angina).

**Figure 2:**
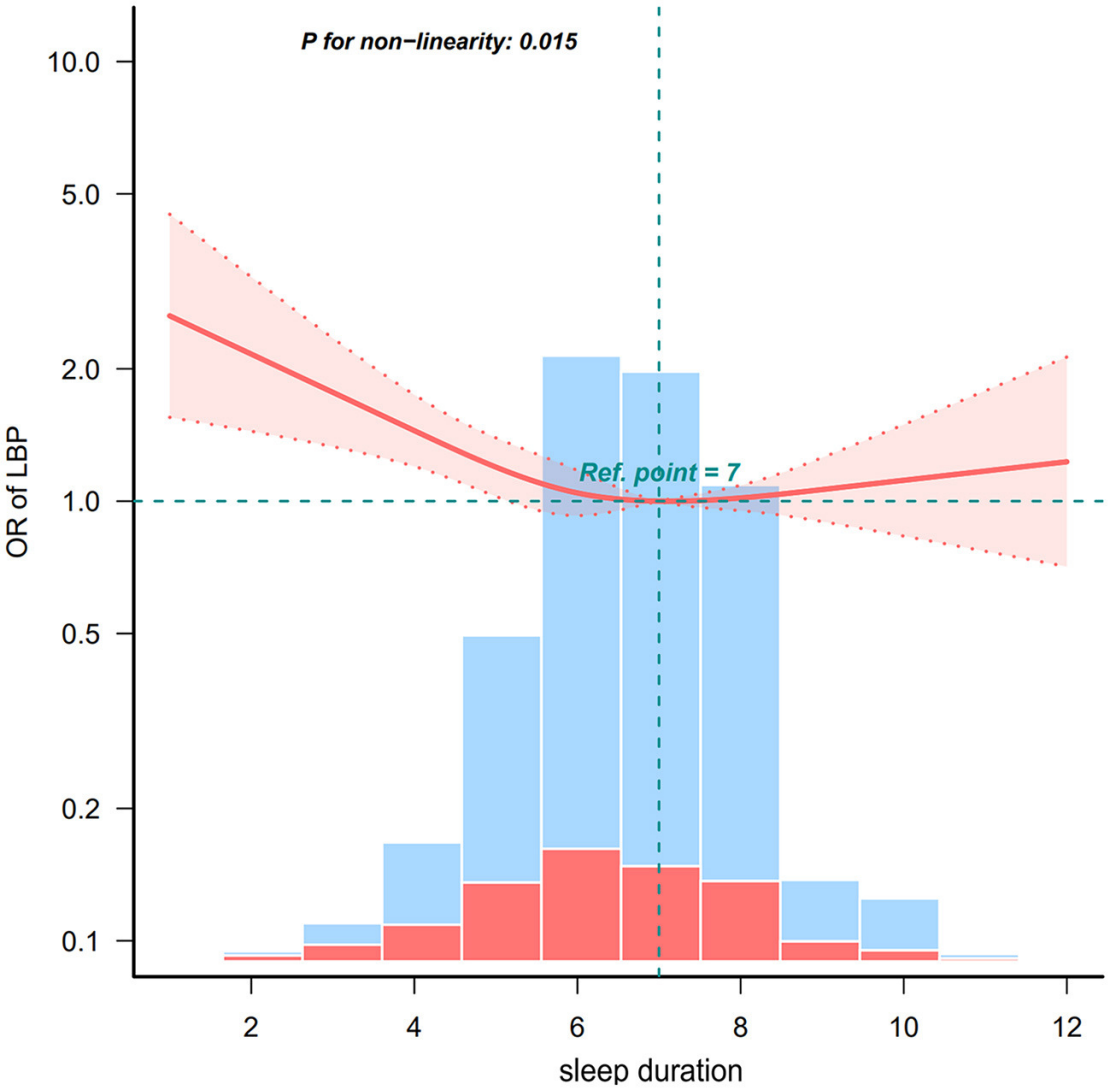
Non-linear relationship between sleep duration and LBP. LBP, low back pain. Solid and dashed lines represent the predicted value and 95% confidence intervals. Adjusted for age, sex, education level, household income, occupation, height, weight, body mass index, smoking status, alcohol consumption, walking day, resistance training day, flexibility exercises day, degree of stress, depression, hypertension, diabetes, stroke, myocardial infarction, angina, and arthritis. Only 99% of the data is presented.

Figure 2 shows the restricted cubic curve spline analysis for fully adjusted model 3, we observed a non-linear relationship between the sleep duration and LBP (*p* for non-linearity = 0.015). The solid line in the figure indicates the predicted risk for the occurrence of LBP, and the dashed line indicates the point-wise 95% confidence interval after adjusting for potential confounding variables, which reveals an L-shaped relationship between sleep duration and the risk of LBP.

The results of further threshold analyses are shown in Table 3, where the risk of LBP was negatively associated with sleep duration when the sleep duration was <6.55 h, and the risk of LBP was reduced by 13.6% for every 1-h increase in sleep duration (OR = 0.864, 95% CI: 0.78–0.96). Nonetheless, no significant association was observed between sleep duration and the risk of LBP when the duration was at least 6.55 h (OR = 1.054, 95% CI: 0.953–1.167).

**Table 3:** Threshold analysis of the relationship of sleep duration with risk of LBP.

**Sleep duration, hour**	**Adjusted Model, OR**	***P*-value**
One-line linear regression model	0.94 (0.9~0.98)	0.004
**Two-piecewise linear regression model**		
Sleep duration <6.55 hours	0.864 (0.78~0.957)	0.005
Sleep duration ≥ 6.55 hours	1.054 (0.953~1.167)	0.307
Likelihood Ratio test		0.006

OR, odds ratio; CI, confidence interval. Adjusted for sociodemographic (age, sex, education level, household income, and occupation), height, weight, body mass index, smoking status, alcohol consumption, walking day, resistance training day, flexibility exercises day, degree of stress, depression, hypertension, diabetes, stroke, myocardial infarction, angina, arthritis. Only 99.9% of the data is shown.

### 3.3 Subgroup analyses

**Figure 3:**
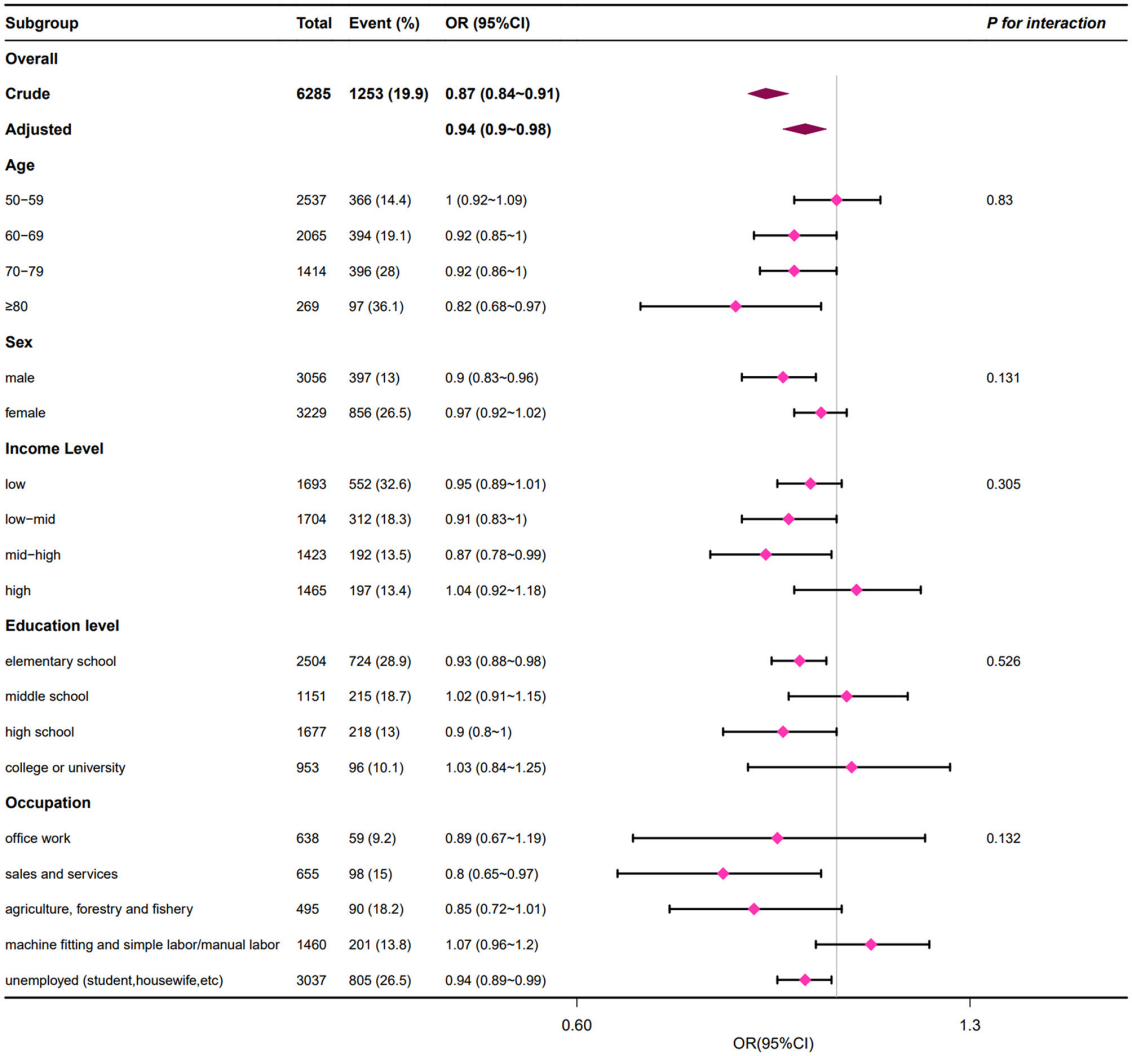
Subgroup analyses of associations of sleep duration with LBP. LBP, low back pain. Adjusted for age, sex, education level, household income, occupation, height, weight, body mass index, smoking status, alcohol consumption, walking day, resistance training day, flexibility exercises day, degree of stress, depression, hypertension, diabetes, stroke, myocardial infarction, angina, and arthritis.

The results of the stratified analysis are shown in Figure 3, which reveals that the relationship between sleep duration and LBP remained stable among different subgroups. We can find that none of the variables of age (50–59, 60–69, 70–79, and ≥80 years), sex (female and male), household income (low, low-mid, mid-high, and high), education (elementary school, middle school, high school, college, or university), and occupation (office work, sales and service, agriculture, forestry and fishery, machine fitting, and simple labor/manual labor, unemployed such as student or housewife) significantly interacted with the relationship between sleep duration and LBP (all *p* for interaction > 0.05).

## 4 Discussion

We used the data from KNHANES to conduct the nationally representative cross-sectional study. After adjusting for covariates, our results showed a significant and independent association between sleep duration and the risk of LBP among adults in Korea aged fifty and above. To our knowledge, our study first revealed that there is an L-curve relationship between sleep duration and risk of LBP. When the sleep duration is <6.55 h, the risk of LBP decreases by 13.6% for each additional hour of sleep. However, when the sleep duration is 6.55 h or longer, further increments in sleep duration do not result in a reduced risk of experiencing LBP. This result was consistent across subgroups defined by age, sex, income level, education level, and occupation.

Previous studies have concentrated on examining the relationship between sleep disorders and LBP (32, 33). The specific dose-response relationship between sleep duration and risk of LBP has been less explored. The findings of this study revealed that individuals suffering from LBP exhibited a markedly reduced sleep duration compared to those without LBP, which may indicate a potential prevalence of sleep deprivation among the LBP patients. After we adjusted for multiple covariates from the biological, sociological, and psychological domains, in-depth analyses using multivariate logistic regression models consistently demonstrated a strong correlation between longer sleep duration and a reduced risk of LBP, thus revealing that adequate sleep may be an independent protective factor against LBP. Recent research findings have unveiled a potential bidirectional mechanism between LBP and sleep disorder (34). On one hand, symptoms of LBP may precipitate sleep disorders; on the other hand, the persistent presence of sleep disorder may exacerbate the pain experience of individuals suffering from LBP (35, 36). In view of this, there remains a meaningful imperative to conduct in-depth research into the interplay between sleep duration and the risk of LBP occurrence.

Sufficient sleep is beneficial in the prevention and treatment of diseases such as stroke, diabetes, and depression (37, 38). However, this study found that the risk of LBP does not appear to be consistently reduced by longer sleep duration. Specifically, the risk of LBP declined with increasing sleep duration only when sleep duration was <6.55 h. The National Institutes of Health (NIH) recommends that adults need 7–8 h of sleep per day10. The natural aging process is often accompanied by a decline in the total sleep duration, which may adversely affect the musculoskeletal system's ability to recuperate, thereby increasing the propensity for LBP to manifest (36, 39, 40). The results of this research suggest that to minimize the risk of LBP in Korea adults aged 50 years and older, it is recommended to secure no <6.55 h of daily sleep. This finding provided a scientific rationale for employing sleep intervention strategies to prevent and manage LBP issues.

Exploration of subgroup analysis data within clinical research is crucial for a more nuanced comprehension of the intricate relationship between exposure variables and outcome variables, which enhances the interpretability of study findings (41). This study revealed that among individuals aged 50 and above, across various age brackets, genders, income levels, educational backgrounds, and occupational categories, there exists a consistent inverse correlation between sleep duration and the risk of LBP. This discovery validated the extensive generalizability of the outcomes across diverse populations. Given the limited attention to the Korean demographic in previous research, the current study analyzed data from KNHANES with the goal of achieving a nationally representative assessment. Consequently, the findings of our study are projected to be broadly relevant and extend to the older adult in Korean, specifically those aged 50 and above. To accurately determine the causal association between sleep duration and LBP, there is a necessity for an increased focus on conducting more prospective longitudinal studies in future research.

Although the specific mechanism between sleep duration and the risk of LBP have yet to be fully elucidated through additional research, current evidence amassed is sufficient to partially explain the findings of this study. Firstly, some studies indicated that insufficient sleep may contribute to the onset and chronicity of LBP through alterations in brain activity, which reduced the pain threshold and impaired cognitive pain processing capabilities (42). Moreover, Krause's study revealed that sleep deprivation intensifies the pain responsiveness in the primary sensory areas of the cerebral cortex, while concurrently reducing the functional activity within other regions involved in pain modulation, such as the striatum and insula (43). Further findings validated that sleep deprivation expands the range for categorizing stimuli as pain, specifically by lowering pain thresholds. Besides, multiple studies have revealed that inflammatory processes may play a significant role in the cycles of pain and sleep disorder. Heffner et al. (44) observed a correlation between reduced sleep quality and elevated IL-6 levels in adults afflicted with LBP. Additionally, the study implicated both diminished sleep quality and increased IL-6 as determinants significantly associated with the severity of pain as reported by the participants (44). The finding indicated that the inflammatory response within individuals with LBP and comorbid sleep disorders may indirectly influence the progression and persistence of pain. A previous systematic review and meta-analysis found that sleep disorder was associated with elevated levels of C-reactive protein (CRP) and IL-6 (45). Proinflammatory cytokines promote disc degeneration by augmenting matrix breakdown and recruiting immune cells to discal tissues (46). Accelerated disc degeneration may trigger LBP. Proinflammatory mediators can exert influences on neurotransmitter systems and neural circuits responsible for mood regulation, arousal, and motor activity, thereby potentially precipitating sleep disturbances (47). However, in the same meta-analysis it was also noted that the shorter sleep duration, but not the extreme of short sleep, was associated with higher levels of CRP but not IL-6 (45). The potential impact of systemic inflammatory markers on the association between sleep duration and LBP requires further researches.

The clinical implications of our study are centered on the significant association between sleep duration and the risk of LBP, particularly for the older adult. By identifying an optimal sleep duration threshold of approximately 6.55 h per day, our findings provide a clear target for clinical recommendations, advocating for sleep hygiene as a preventive measure in LBP management. The non-linear relationship between sleep and LBP risk suggests a focus on achieving adequate sleep without exceeding the threshold, where additional sleep offers no further protective effect. This insight has direct applications in clinical practice, where healthcare providers can assess and improve patients' sleep as part of routine LBP evaluations and treatment plans. Furthermore, our results highlight the necessity for integrated care approaches that consider sleep management as an integral component of LBP therapy, potentially involving a multidisciplinary team. Lastly, our findings call for additional research into the mechanisms connecting sleep and LBP, which could lead to more targeted interventions and improved patient outcomes. Collectively, these implications contribute to a more comprehensive understanding of LBP management, emphasizing the role of sleep as a modifiable risk factor and the need for further investigation into its underlying pathways.

This study exhibits several distinct advantages: Firstly, the study is underpinned by KNHANES, a large-scale, nationally representative population study. Consequently, the substantial sample size ensures the reliability and robustness of the findings. Subsequently, threshold analyses were conducted to further elucidate the L-shaped relationship between sleep duration and the incidence of LBP. Ultimately, stratified subgroup analyses were conducted, revealing no significant interactions with other factors.

Certainly, the current study is not without its inherent limitations. Firstly, sleep duration was defined through patients' self-reported outcomes, which may be subject to recall bias. Similarly, the accuracy of diagnoses for LBP could introduce bias into the study findings. What's more, the cross-sectional nature of our study precludes the inference of causality between short sleep duration and LBP. Additionally, this study faced limitations inherent to the data available in public databases, with a shortfall in adjusting for critical confounders, including a history of lumbar disc herniation, recurrence rates, and pain severity. Furthermore, our study focused on the duration of sleep but did not provide an in-depth assessment of sleep disorders such as insomnia. Future studies that incorporate a more nuanced examination of sleep disorders in relation to LBP could significantly enhance our understanding of their interplay, potentially leading to more targeted and effective clinical interventions for managing sleep disturbances in patients with LBP.

## 5 Conclusion

In conclusion, our study showed that an increase in sleep duration was inversely associated with a decreased risk of LBP when sleep duration was <6.55 h range in various categories of people over 50 years old in Korea. Therefore, adequate sleep duration may be an important factor in the prevention of LBP in older adults.

## Data Availability

Publicly available datasets were analyzed in this study. This data can be found here: https://knhanes.kdca.go.kr/knhanes/sub03/sub03_02_05.do.
