# Supplementary material for: Sleeping <6.55 h per day was associated with a higher risk of low back pain in adults aged over 50 years: a Korean nationwide cross-sectional study
Source: Front Public Health. 2024 Sep 20;12:1429495. doi: 10.3389/fpubh.2024.1429495 (PMC11449761; doi:10.3389/fpubh.2024.1429495)
Supplement: Supplementary file 1 [file Data_Sheet_1.PDF]

Supplementary Material

Supplementary Content

Supplementary Table S1 The basic characteristics of the excluded (data missing, n = 13317) and included (data completed, n =6285) participants.

Supplementary Table S2 KNHANES 2010-2015 participant characteristics stratified by sleep duration status.

Supplementary Table S3 Univariate analysis for the low back pain.

Table S1 The basic characteristics of the excluded and included participants

Variables	Total (n = 19602)	Exclude (n = 13317)	Include (n = 6285)	*p*
**LBP, n (%)**				< 0.001
no	13267 (76.0)	8235 (73.7)	5032 (80.1)	
yes	4199 (24.0)	2946 (26.3)	1253 (19.9)	
**Sleep(hour), Mean ± SD**	6.6 ± 1.5	6.6 ± 1.6	6.6 ± 1.5	0.568
**Age(year), Mean ± SD**	64.3 ± 9.1	64.8 ± 9.3	63.1 ± 8.7	< 0.001
**Height(cm), Mean ± SD**	159.0 ± 8.9	158.2 ± 9.0	160.4 ± 8.7	< 0.001
**Weight(cm), Mean ± SD**	60.8 ± 10.2	60.2 ± 10.2	61.9 ± 10.2	< 0.001
**Body mass index (kg/m2), Mean ± SD**	24.0 ± 3.1	24.0 ± 3.2	24.0 ± 3.1	0.294
**Sex, n (%)**				< 0.001
male	8398 (42.8)	5342 (40.1)	3056 (48.6)	
female	11204 (57.2)	7975 (59.9)	3229 (51.4)	
**Household income, n (%)**				< 0.001
low	6134 (31.7)	4441 (34)	1693 (26.9)	
low-mid	5095 (26.3)	3391 (25.9)	1704 (27.1)	
mid-high	4003 (20.7)	2580 (19.7)	1423 (22.6)	
high	4123 (21.3)	2658 (20.3)	1465 (23.3)	
**Education level, n (%)**				< 0.001
elementary school	8057 (46.4)	5553 (50.1)	2504 (39.8)	
middle school	3001 (17.3)	1850 (16.7)	1151 (18.3)	
high school	4137 (23.8)	2460 (22.2)	1677 (26.7)	
college or university	2177 (12.5)	1224 (11)	953 (15.2)	
**Occupation, n (%)**				< 0.001
office work	1468 (8.5)	830 (7.5)	638 (10.2)	
sales and services	1723 (9.9)	1068 (9.6)	655 (10.4)	
agriculture, forestry and fishery	1984 (11.4)	1489 (13.4)	495 (7.9)	
machine fitting and simple labor/manual labor	3524 (20.3)	2064 (18.6)	1460 (23.2)	
unemployed (student, housewife, etc.)	8660 (49.9)	5623 (50.8)	3037 (48.3)	
**Walking, n (%)**				0.208
none	3711 (21.4)	2418 (21.9)	1293 (20.6)	
1–2 day/week	2602 (15.0)	1663 (15.1)	939 (14.9)	
3–4 day/week	3415 (19.7)	2158 (19.5)	1257 (20)	
≥5 day/week	7605 (43.9)	4809 (43.5)	2796 (44.5)	
**Resistance training, n (%)**				< 0.001
none	13274 (76.5)	8525 (77)	4749 (75.6)	
1–2 day/week	1497 (8.6)	1000 (9)	497 (7.9)	
3–4 day/week	1233 (7.1)	789 (7.1)	444 (7.1)	
≥5 day/week	1355 (7.8)	760 (6.9)	595 (9.5)	
**Flexibility exercises, n (%)**				< 0.001
none	8519 (49.1)	5670 (51.2)	2849 (45.3)	
1–2 day/week	2587 (14.9)	1680 (15.2)	907 (14.4)	
3–4 day/week	2602 (15.0)	1616 (14.6)	986 (15.7)	
≥5 day/week	3650 (21.0)	2107 (19)	1543 (24.6)	
**Smoking status, n (%)**				< 0.001

non/ex-smoker	14841 (84.6)	9660 (85.8)	5181 (82.4)	
current smoker	2703 (15.4)	1599 (14.2)	1104 (17.6)	
**Alcohol consumption, n (%)**				< 0.001
none	3342 (24.3)	1731 (23.1)	1611 (25.6)	
≤1 drink/month	4403 (32.0)	2421 (32.3)	1982 (31.5)	
2 drinks/month to 3 drinks/week	4521 (32.8)	2465 (32.9)	2056 (32.7)	
≥4 drinks/week	1504 (10.9)	868 (11.6)	636 (10.1)	
**Degree of Stress, n (%)**				< 0.001
none	4245 (24.2)	2706 (24.1)	1539 (24.5)	
mild	9694 (55.3)	6104 (54.3)	3590 (57.1)	
moderate	2889 (16.5)	1962 (17.4)	927 (14.7)	
severe	704 (4.0)	475 (4.2)	229 (3.6)	
**Depression, n (%)**				< 0.001
no	8864 (89.8)	2967 (82.8)	5897 (93.8)	
yes	1003 (10.2)	615 (17.2)	388 (6.2)	
**Hypertension, n (%)**				< 0.001
no	5062 (42.3)	1079 (18.9)	3983 (63.4)	
yes	6918 (57.7)	4616 (81.1)	2302 (36.6)	
**Diabetes, n (%)**				< 0.001
no	6945 (72.7)	1596 (48.8)	5349 (85.1)	
yes	2611 (27.3)	1675 (51.2)	936 (14.9)	
**Dyslipidemia, n (%)**				< 0.001
no	6402 (64.5)	1528 (41.9)	4874 (77.5)	
yes	3526 (35.5)	2115 (58.1)	1411 (22.5)	
**Stroke, n (%)**				< 0.001
no	7798 (91.6)	1793 (80.6)	6005 (95.5)	
yes	712 (8.4)	432 (19.4)	280 (4.5)	
**Myocardial infarction, n (%)**				< 0.001
no	8042 (96.6)	1862 (91.5)	6180 (98.3)	
yes	279 (3.4)	174 (8.5)	105 (1.7)	
**Angina, n (%)**				< 0.001
no	7930 (92.6)	1830 (80.4)	6100 (97.1)	
yes	630 (7.4)	445 (19.6)	185 (2.9)	
**Arthritis, n (%)**				< 0.001
no	6875 (65.0)	1771 (41.2)	5104 (81.2)	
yes	3706 (35.0)	2525 (58.8)	1181 (18.8)	

SD, standard deviation

Table S2 KNHANES 2010-2015 participant characteristics stratified by sleep duration status.

Variables	Sleep Duration, hour						
	Total (n = 6285)	Q1(≤5h) (n = 1336)	Q2(6h) (n = 1635)	Q3(7h) (n = 1592)	Q4(8h) (n = 1286)	Q5(≥9h) (n = 436)	*p*
**Low back pain, n (%)**							< 0.001
yes	1253(19.9)	374(28)	304(18.6)	257(16.1)	217(16.9)	101(23.2)	
no	5031(80.1)	962(72)	1331(81.4)	1335(83.9)	1069(83.1)	335(76.8)	
**Age(year), Mean ± SD**	63.1 ± 8.7	64.9 ± 9.0	62.4 ± 8.5	62.1 ± 8.4	62.7 ± 8.5	65.9 ± 9.2	< 0.001
**Height(cm), Mean ± SD**	160.4 ± 8.7	158.1 ± 8.8	160.9 ± 8.5	161.1 ± 8.7	161.2 ± 8.2	160.1 ± 9.1	< 0.001
**Weight(kg), Mean ± SD**	61.9 ± 10.2	60.0 ± 10.1	62.5 ± 10.3	62.5 ± 10.4	62.5 ± 9.1	62.1 ± 11.2	< 0.001
**Body mass index (kg/m^2), Mean ± SD**	24.0 ± 3.1	24.0 ± 3.2	24.1 ± 3.1	24.0 ± 3.1	24.0 ± 2.9	24.2 ± 3.6	0.811
**Sex, n (%)**							< 0.001
male	3056 (48.6)	508 (38)	827 (50.6)	807 (50.7)	686 (53.3)	228 (52.3)	
female	3229 (51.4)	828 (62)	808 (49.4)	785 (49.3)	600 (46.7)	208 (47.7)	
**Household income, n (%)**							< 0.001
low	1693 (26.9)	473 (35.4)	393 (24)	331 (20.8)	320 (24.9)	176 (40.4)	
low-mid	1704 (27.1)	400 (29.9)	425 (26)	416 (26.1)	348 (27.1)	115 (26.4)	
mid-high	1423 (22.6)	265 (19.8)	384 (23.5)	381 (23.9)	314 (24.4)	79 (18.1)	
high	1465 (23.3)	198 (14.8)	433 (26.5)	464 (29.1)	304 (23.6)	66 (15.1)	
**Education level, n (%)**							< 0.001
elementary school	2504 (39.8)	748 (56)	568 (34.7)	511 (32.1)	458 (35.6)	219 (50.2)	
middle school	1151 (18.3)	236 (17.7)	294 (18)	266 (16.7)	269 (20.9)	86 (19.7)	
high school	1677 (26.7)	225 (16.8)	477 (29.2)	514 (32.3)	363 (28.2)	98 (22.5)	
college or university	953 (15.2)	127 (9.5)	296 (18.1)	301 (18.9)	196 (15.2)	33 (7.6)	
**Occupation, n (%)**							< 0.001
office work	638 (10.2)	100 (7.5)	198 (12.1)	201 (12.6)	118 (9.2)	21 (4.8)	
sales and services	655 (10.4)	124 (9.3)	181 (11.1)	202 (12.7)	125 (9.7)	23 (5.3)	
agriculture, forestry and fishery	495 (7.9)	87 (6.5)	126 (7.7)	106 (6.7)	127 (9.9)	49 (11.2)	
machine fitting and simple labor/manual labor	1460 (23.2)	291 (21.8)	391 (23.9)	385 (24.2)	319 (24.8)	74 (17)	
unemployed (Student, housewife, etc.)	3037 (48.3)	734 (54.9)	739 (45.2)	698 (43.8)	597 (46.4)	269 (61.7)	

**Walking, n (%)**							< 0.001
none	1293 (20.6)	320 (24)	320 (19.6)	267 (16.8)	258 (20.1)	128 (29.4)	
1–2 day/week	939 (14.9)	187 (14)	252 (15.4)	253 (15.9)	184 (14.3)	63 (14.4)	
3–4 day/week	1257 (20.0)	229 (17.1)	347 (21.2)	350 (22)	262 (20.4)	69 (15.8)	
⩾5 day/week	2796 (44.5)	600 (44.9)	716 (43.8)	722 (45.4)	582 (45.3)	176 (40.4)	
**Resistance training, n (%)**							< 0.001
none	4749 (75.6)	1070 (80.1)	1207 (73.8)	1142 (71.7)	979 (76.1)	351 (80.5)	
1–2 day/week	497 (7.9)	91 (6.8)	132 (8.1)	149 (9.4)	94 (7.3)	31 (7.1)	
3–4 day/week	444 (7.1)	74 (5.5)	124 (7.6)	129 (8.1)	97 (7.5)	20 (4.6)	
⩾5 day/week	595 (9.5)	101 (7.6)	172 (10.5)	172 (10.8)	116 (9)	34 (7.8)	
**Flexibility exercises, n (%)**							< 0.001
none	2849 (45.3)	668 (50)	688 (42.1)	618 (38.8)	597 (46.4)	278 (63.8)	
1–2 day/week	907 (14.4)	149 (11.2)	244 (14.9)	285 (17.9)	189 (14.7)	40 (9.2)	
3–4 day/week	986 (15.7)	200 (15)	271 (16.6)	261 (16.4)	194 (15.1)	60 (13.8)	
⩾5 day/week	1543 (24.6)	319 (23.9)	432 (26.4)	428 (26.9)	306 (23.8)	58 (13.3)	
**Smoking status, n (%)**							< 0.001
non/ex-smoker	5181 (82.4)	1142 (85.5)	1335 (81.7)	1345 (84.5)	1023 (79.5)	336 (77.1)	
current smoker	1104 (17.6)	194 (14.5)	300 (18.3)	247 (15.5)	263 (20.5)	100 (22.9)	
**Alcohol consumption, n (%)**							< 0.001
none	1611 (25.6)	405 (30.3)	415 (25.4)	350 (22)	309 (24)	132 (30.3)	
⩽1 drink/month	1982 (31.5)	404 (30.2)	495 (30.3)	549 (34.5)	401 (31.2)	133 (30.5)	
2 drinks/month to 3 drinks/week	2056 (32.7)	370 (27.7)	587 (35.9)	538 (33.8)	441 (34.3)	120 (27.5)	
⩾4 drinks/week	636 (10.1)	157 (11.8)	138 (8.4)	155 (9.7)	135 (10.5)	51 (11.7)	
**Degree of Stress, n (%)**							< 0.001
none	1539 (24.5)	320 (24)	373 (22.8)	378 (23.7)	330 (25.7)	138 (31.7)	
mild	3590 (57.1)	655 (49)	965 (59)	979 (61.5)	775 (60.3)	216 (49.5)	
moderate	927 (14.7)	279 (20.9)	247 (15.1)	195 (12.2)	140 (10.9)	66 (15.1)	
severe	229 (3.6)	82 (6.1)	50 (3.1)	40 (2.5)	41 (3.2)	16 (3.7)	
**Depression, n (%)**							< 0.001
no	5897 (93.8)	1223 (91.5)	1536 (93.9)	1503 (94.4)	1228 (95.5)	407 (93.3)	
yes	388 (6.2)	113 (8.5)	99 (6.1)	89 (5.6)	58 (4.5)	29 (6.7)	
**Hypertension, n (%)**							< 0.001
no	3983 (63.4)	782 (58.5)	1060 (64.8)	1045 (65.6)	846 (65.8)	250 (57.3)	
yes	2302 (36.6)	554 (41.5)	575 (35.2)	547 (34.4)	440 (34.2)	186 (42.7)	

**Diabetes, n (%)**							0.002
no	5349 (85.1)	1121 (83.9)	1422 (87)	1377 (86.5)	1077 (83.7)	352 (80.7)	
yes	936 (14.9)	215 (16.1)	213 (13)	215 (13.5)	209 (16.3)	84 (19.3)	
**Dyslipidemia, n (%)**							< 0.001
no	4874 (77.5)	988 (74)	1266 (77.4)	1237 (77.7)	1046 (81.3)	337 (77.3)	
yes	1411 (22.5)	348 (26)	369 (22.6)	355 (22.3)	240 (18.7)	99 (22.7)	
**Stroke, n (%)**							< 0.001
no	6005 (95.5)	1268 (94.9)	1575 (96.3)	1534 (96.4)	1239 (96.3)	389 (89.2)	
yes	280 (4.5)	68 (5.1)	60 (3.7)	58 (3.6)	47 (3.7)	47 (10.8)	
**Myocardial infarction, n (%)**							0.021
no	6180 (98.3)	1311 (98.1)	1598 (97.7)	1569 (98.6)	1276 (99.2)	426 (97.7)	
yes	105 (1.7)	25 (1.9)	37 (2.3)	23 (1.4)	10 (0.8)	10 (2.3)	
**Angina, n (%)**							0.771
no	6100 (97.1)	1299 (97.2)	1589 (97.2)	1548 (97.2)	1241 (96.5)	423 (97)	
yes	185 (2.9)	37 (2.8)	46 (2.8)	44 (2.8)	45 (3.5)	13 (3)	
**Arthritis, n (%)**							< 0.001
no	5104 (81.2)	1003 (75.1)	1313 (80.3)	1348 (84.7)	1100 (85.5)	340 (78)	
yes	1181 (18.8)	333 (24.9)	322 (19.7)	244 (15.3)	186 (14.5)	96 (22)	

SD, standard deviation.

able S3. Univariate analysis for the low back pain.

Variable	OR_95CI	*P*
**Sleep duration(hour), Mean ± SD**	0.87 (0.84~0.91)	<0.001
**Age(year), Mean ± SD**	1.05 (1.04~1.05)	<0.001
**Height(cm), Mean ± SD**	0.94 (0.93~0.95)	<0.001
**Weight(kg), Mean ± SD**	0.97 (0.96~0.98)	<0.001
**BMI (kg/m2), Mean ± SD**	1.01 (0.99~1.03)	0.288
**Sex, n (%)**		
male	1(reference)	
female	2.42 (2.12~2.75)	<0.001
**Household income, n (%)**		
low	1(reference)	
low-mid	0.46 (0.4~0.54)	<0.001
mid-high	0.32 (0.27~0.39)	<0.001
high	0.32 (0.27~0.38)	<0.001
**Education level, n (%)**		
elementary school	1(reference)	
middle school	0.56 (0.48~0.67)	<0.001
high school	0.37 (0.31~0.43)	<0.001
college or university	0.28 (0.22~0.35)	<0.001
**Occupation, n (%)**		
office work	1(reference)	
sales and services	1.73 (1.22~2.43)	0.002
agriculture, forestry and fishery	2.18 (1.53~3.1)	<0.001
machine fitting and simple labor/manual labor	1.57 (1.15~2.13)	0.004
unemployed (Student, housewife, etc.)	3.54 (2.68~4.68)	<0.001
**Walking, n (%)**		
none	1(reference)	
1–2 day/week	0.76 (0.62~0.94)	0.009
3–4 day/week	0.76 (0.63~0.92)	0.004
≥5 day/week	0.69 (0.58~0.8)	<0.001
**Resistance training, n (%)**		
none	1(reference)	
1–2 day/week	0.67 (0.52~0.86)	0.002
3–4 day/week	0.69 (0.53~0.89)	0.005
≥5 day/week	0.38 (0.29~0.51)	<0.001
**Flexibility exercises, n (%)**		
none	1(reference)	
1–2 day/week	0.77 (0.63~0.93)	0.007
3–4 day/week	0.92 (0.77~1.1)	0.35
≥5 day/week	0.66 (0.56~0.78)	<0.001
**Smoking status, n (%)**		
non/ex-smoker	1(reference)	
current smoker	0.87 (0.73~1.03)	0.096
**Alcohol consumption, n (%)**		
none	1(reference)	

≤1 drink/month	0.89 (0.76~1.03)	0.126
2 drinks/month to 3 drinks/week	0.57 (0.48~0.67)	<0.001
≥4 drinks/week	0.66 (0.52~0.84)	0.001
**Degree of Stress, n (%)**		
none	1(reference)	
mild	1.24 (1.06~1.46)	0.008
moderate	2.44 (2~2.97)	<0.001
severe	2.58 (1.89~3.52)	<0.001
**Depression, n (%)**		
no	1(reference)	
yes	2.91 (2.35~3.6)	<0.001
**Hypertension, n (%)**		
no	1(reference)	
yes	1.39 (1.22~1.57)	<0.001
**Diabetes, n (%)**		
no	1(reference)	
yes	1.52 (1.29~1.78)	<0.001
**Dyslipidemia, n (%)**		
no	1(reference)	
yes	1.5 (1.31~1.73)	<0.001
**Stroke, n (%)**		
no	1(reference)	
yes	2.22 (1.72~2.87)	<0.001
**Myocardial infarction, n (%)**		
no	1(reference)	
yes	1.26 (0.8~1.98)	0.317
**Angina, n (%)**		
no	1(reference)	
yes	1.87 (1.37~2.57)	<0.001
**Arthritis, n (%)**		
no	1(reference)	
yes	3.17 (2.75~3.64)	<0.001

OR, odds ratio; CI, confidence interval; BMI, body mass index;
